# Analysis of Fluctuation in Cerebral Venous Oxygenation Using MR Imaging: Quantitative Evaluation of Vasomotor Function of Arterioles

**DOI:** 10.2463/mrms.mp.2015-0156

**Published:** 2016-04-28

**Authors:** Minghui Tang, Keigo Nishi, Toru Yamamoto

**Affiliations:** 1 Graduate School of Health Sciences, Hokkaido University; 2 Division of Biomedical Engineering and Science, Faculty of Health Sciences, Hokkaido University, Kita 12 Nishi 5, Kita-ku, Sapporo, Hokkaido 060-0812, Japan

**Keywords:** blood oxygenation, carbon dioxide, arteriole, vasomotor

## Abstract

**Purpose:**

Cerebral arteriolar vasomotor function plays an important role in brain health. Since respiratory changes in the partial arterial pressure of CO_2_ (PaCO_2_) cause arterioles to vasodilate or vasoconstrict, resting-state arteriolar vasomotion results in the fluctuation of venous blood oxygenation, which can be monitored by observing magnetic resonance (MR) signals. Focusing on the superior sagittal sinus as the largest cerebral vein, we developed a method to elucidate the respiratory fluctuation of cerebral venous oxygenation that may reflect the vasomotor function.

**Methods:**

Single slices of varying thickness (7–15 mm) taken perpendicular to the superior sagittal sinus of five volunteers were imaged by spin-echo echo-planar imaging using a 1.5-T MR system. The time series of the signal intensity at the superior sagittal sinus was Fourier-transformed, and the spectral fluctuation intensity (SFI) at respiratory frequency was obtained. The amplitude of the respiratory fluctuation in the cerebral venous oxygenation was calculated from the gradient of the relation between the SFI and the average signal intensity, which increased proportionally with an increase in slice thickness. The amplitude of the fluctuation in cerebral venous oxygenation at low (<0.1 Hz) and cardiac pulsation frequencies was also calculated for comparison with the respiratory fluctuation.

**Results:**

The amplitude of respiratory fluctuation in the cerebral venous oxygenation was quantified as 1.2%, demonstrating the validity of our method via the highest significant correlation (*r* = 0.82) in the plot of SFI and average signal intensities; the correlations at low and cardiac pulsation frequencies were 0.60 and 0.63, respectively.

**Conclusion:**

We have successfully demonstrated cerebral venous oxygenation fluctuation at respiratory frequencies in the resting state. This fluctuation was non-invasively evaluated as 1.2%, representing the control value for the arteriolar vasomotor function of a healthy human.

## Introduction

Cerebral arteriolar vasomotor function, that is, the vasodilation and vasoconstriction properties of arterioles, plays an important role in brain health. Dysfunction or impairment of this arteriolar vasomotor function may cause cerebral ischemia, which will augment the accumulation of neurotoxic factors such as amyloid-beta.^1^ This accumulation results in dementia such as Alzheimer’s disease.^2^ From this pathological consequence, the deterioration of the arteriolar vasomotor function may be an early marker of dementia.^3,4^ Several methods have been developed to observe the arteriolar vasomotor function measured by a response to a vasodilator agent. Using the transcranial Doppler test, the blood flow velocity in the middle cerebral artery was measured with and without a vasodilator such as Diamox or carbon dioxide (CO_2_).^5,6^ Single photon emission computed tomography successfully measured changes in the cerebral blood flow (CBF) via CO_2_ challenges.^7^ Further, to directly map the arteriolar vasomotor function, a mapping technique called cerebral vascular reactivity (CVR) was developed using magnetic resonance imaging (MRI).^8^ Although human arteriolar vasomotor function has been observed by these methods, all techniques developed until now are invasive in terms of the need for drug administration or CO_2_ inhalation, which can be challenging for patients in clinical practice. To obtain information about the arteriolar vasomotion function non-invasively, we focused on the fluctuations of the magnetic resonance (MR) signals of venous blood in the resting state.

CO_2_ is a vasodilator that drives the arteriolar vasomotion and modulates the CBF, resulting in the fluctuation of venous oxygenation. CO_2_ in the blood is quantified by the arterial partial pressure of CO_2_ (PaCO_2_), and this fluctuates at respiratory frequencies of around 0.3 Hz.^9^ The fluctuation of venous blood oxygenation at respiratory frequencies reflects the arteriolar vasomotion induced by changes in respiratory CO_2_.^10^

The MR signal of the blood depends on blood oxygenation, because the transverse relaxation time increases with an increase in the blood oxygenation level.^11^ As it has been reported that the MR signal from the superior sagittal sinus, which is the largest cerebral vein, reflects the cerebral venous oxygenation,^12^ fluctuations in the MR signal from the superior sagittal sinus should reflect the changes in cerebral venous oxygenation. To observe the fluctuation of cerebral venous oxygenation, we obtained time series of the MR signal in the superior sagittal sinus using successive rapid MR imaging of the brain across the superior sagittal sinus. Spectral analysis of these time series data yielded the respiratory fluctuation of the MR signal from venous blood in the superior sagittal sinus. However, this fluctuation was also influenced by changes in the blood flow velocity. To elucidate the fluctuation of venous blood oxygenation, we developed a method to differentiate the MR signal fluctuation due to blood oxygenation and blood flow. We applied this non-invasive method without using drug administration/CO_2_ inhalation to healthy volunteers, and derived the respiratory fluctuation in cerebral venous oxygenation that reflects healthy vasomotor function of the arterioles based on the vasomotion due to respiratory CO_2_ changes in the resting state. Because CBF also fluctuates at low frequencies (<0.1 Hz), reflecting default mode brain function,^13^ and at the cardiac frequency, the amplitude of the fluctuation in cerebral venous oxygenation at those frequencies was also spectrally calculated for comparison with the respiratory fluctuation.

## Theory

### Spectral analysis of MR signal time series

The fluctuation in the time series data *S*(*n*), where *n* is the temporal sampling point number, can be described using its standard deviation (*SD*), i.e.,

[1] $$\mathrm{SD}=\sqrt{\frac{\sum_{n=1}^{N}{\left(S(n)-S_{\text{average}}\right)}^{2}}{N},}$$

where *S*_average_ is the mean value of *S*(*n*) and *N* is the total number of data points. We define *S*(*n*) − *S*_average_ as *x*(*n*). According to Parseval’s theorem, the relationship between *x*(*n*) and its discrete Fourier transform *X*(*m*) is expressed as

[2] $$\sum_{n=1}^{N}{\left|x(n)\right|}^{2}=\frac{1}{N}\;\sum_{m=1}^{N}{\left|X(m)\right|}^{2},$$

where *m* is a spectral frequency point number and |*X*(*m*)|^2^ is the power spectrum. Combining Eqs. 1[1] and [2], *SD* can be transformed into

[3] $$\mathrm{SD}=\frac{1}{N}\sqrt{\sum_{m=1}^{N}{\left|X(m)\right|}^{2},}$$

To represent the standard deviation of the temporal fluctuation component in the MR signal composed of fluctuations in the frequency range [*ω*_1_, *ω*_2_], we defined the spectral fluctuation intensity (*SFI*) as

[4] $$\mathrm{SFI}=\frac{1}{N}\sqrt{2\times \sum_{m=m_{1}}^{m_{2}}{\left|X(\omega )\right|}^{2},}$$

where *m*_1_ and *m*_2_ are the spectral frequency point numbers for *ω*_1_, *ω*_2_, respectively. As *X*(*m*) is a transform of the real signal *x*(*n*), |*X*(*m*)|^2^ is symmetric about the zero frequency. Therefore, the summation of the power spectral intensity at positive frequencies is twice that given by Eq. [4] .

### Analysis of blood MR signal fluctuation

**Fig 1.**
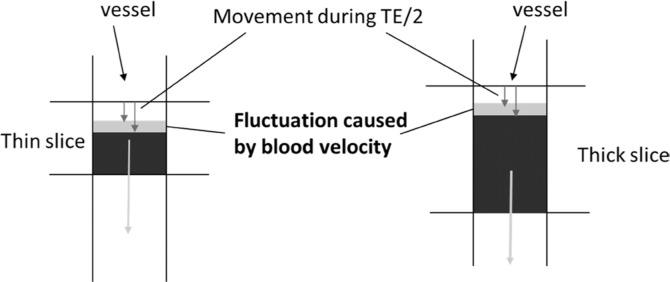
SE–EPI signal of a blood vessel perpendicularly crossing the imaging slice. The blood signal (black blocks) fluctuates due to the variation in blood velocity (gray blocks), which is independent of the slice thickness.

**Fig 2.**
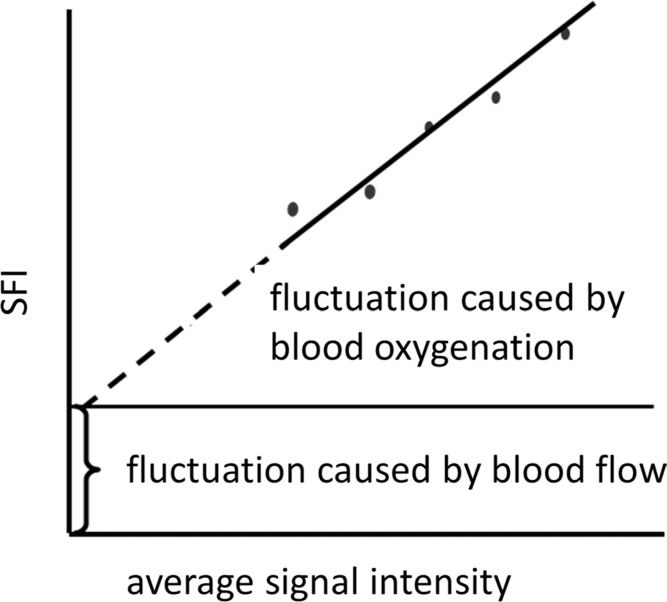
Plot of the spectral fluctuation intensity versus average signal intensity. The average signal intensity increases proportionally with an increase in slice thickness.

We focused on the respiratory fluctuation in the spin-echo echo-planar imaging (SE–EPI) MR signal of venous blood that can be monitored by spectral analysis of the time series images of a single slice perpendicular to the superior sagittal sinus. Because the respiratory fluctuation in the blood MR signal was not only induced by changes in the venous oxygenation level, but also by fluctuation in blood flow velocity due to respiration,^14^ we developed a method to separate the influence of oxygenation and blood flow velocity fluctuation in the temporal MR signal of the venous blood in the superior sagittal sinus. This method employed SE–EPI with different slice thicknesses at the same location across the sinus. Because the SE–EPI MR signal of the blood vessel increases with slice thickness (black blocks in Fig. 1) and the blood signal depends on the blood oxygenation, the MR signal fluctuation due to blood oxygenation increases with slice thickness, which is proportional to the average signal intensity of the blood vessel. In contrast, the amplitude of the fluctuation in blood flow velocity remains constant (gray blocks in Fig. 1) with respect to slice thickness, because the signal fluctuation induced by blood velocity variation is independent of the slice thickness whenever it covers the whole variation area in blood velocity (gray blocks in Fig. 1). The plot of SFI versus average signal intensity shows the fluctuation caused by blood oxygenation (proportional component in Fig. 2) and that caused by the blood velocity (y-intercept in Fig. 2). In this theory, we assumed a constant blood vessel size in the superior sagittal sinus for the different slice thicknesses.

### Evaluation of the respiratory fluctuation in blood oxygenation

The SE MR signal can be expressed as

[5] $$S=C\cdot {\text{e}}^{-R_{2}\cdot TE},$$

where *C* is a proportional constant, *TE* is echo time, and *R*_2_ is a transverse relaxation rate of the blood, expressed as

[6] $$R_{2}=C_{1}\cdot {\left(1-Y\right)}^{2}+C_{2},$$

Where *C*_1_ and *C*_2_ are determined by the magnetic field strength and hematocrit fraction, respectively, and *Y* is the blood oxygenation.^15^ Using Eqs. [5] and [6], we can derive the following equation:

[7] $$\frac{\Delta S}{S}=2C_{1}\cdot (1-Y)\cdot TE\cdot \Delta Y,$$

Where Δ*S* is the MR signal fluctuation caused by the fluctuation in blood oxygenation (Δ*Y*) and *S* is the average signal intensity of the blood. Because Δ*S* is expressed by the SFI (Eq. [4]) and Δ*S/S* is obtained as the gradient of the regression line in the plot of SFI versus the average signal intensity (Fig. 2); the blood oxygenation fluctuation (Δ*Y*) can be calculated from Eq. [7]. In this calculation, we used *Y* = 0.66 as the baseline value of venous blood in healthy human subjects.^16^ We used *C*_1_ = 59 (taken from Eq. [13]);^17^ the value for normal hematocrit (0.40) was also employed.

## Materials and Methods

### Subjects

Five volunteers (male, aged 24 ± 2 years) were participated in this study. All experiments were carried out with the approval of the institutional review board.

### MR imaging

**Fig 3.**
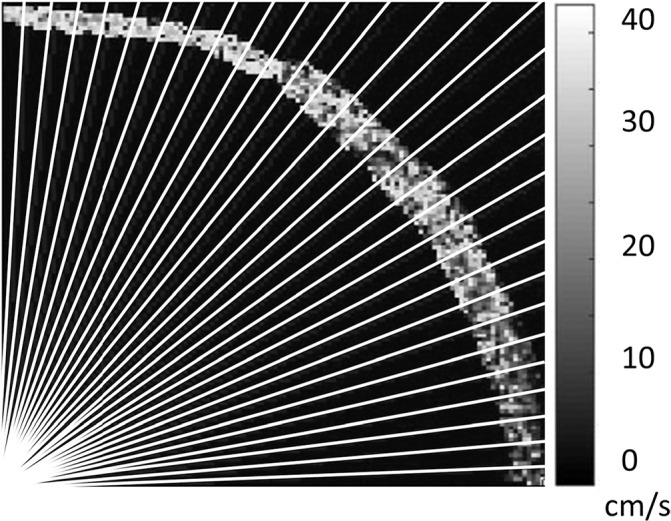
Velocity mapping of the superior sagittal sinus of a volunteer. The white lines represent the boundaries of 30 blocks in which the blood velocity was averaged.

We used a 1.5-T MRI (Signa LX, General Electric). As the theory was based on a constant blood vessel size, that is, consistent blood flow, through all slice thicknesses at the imaged location, we observed the blood velocity along the vessel. The blood velocity mapping of the superior sagittal sinus was performed using 2D fast phase contrast (PC) imaging synchronized with systolic cardiac pulsation monitored by the built-in physiology monitor of the MRI system. Velocity encoding was set at 40 cm/s for the x, y, and z directions. A single slice (thickness = 10 mm, field of view (FOV) = 170 mm × 170 mm, matrix size = 256 × 256) covering the superior sagittal sinus was imaged using a 3-in (∼76 mm) surface coil to maintain a high signal-to-noise ratio. The scan time of this 2D fast PC imaging was <4 min, depending on the individual heart rate. To quantify the velocity changes along the superior sagittal sinus, the velocity mapping was divided into 30 blocks (Fig. 3), and the mean velocity at each block was calculated. The spatial derivative of the blood flow velocity along the blocks on the superior sagittal sinus was obtained to evaluate the consistency of blood velocity through all slice thicknesses at each imaging location.

A single slice perpendicular to the superior sagittal sinus was successively imaged for 45 s using SE–EPI (TE = 30 ms, matrix size = 128 × 128, FOV = 170 mm × 170 mm, bandwidth = 69 kHz) with the same surface coil. The repetition time (TR) was set to 250 ms to give a Nyquist frequency of 2 Hz, covering the cardiac pulsation frequency. The imaging was repeated for various slice thicknesses (7, 9, 11, 13, and 15 mm). The total acquisition time of SE–EPI at each slice location was ∼5 min. To observe the positional influence on the results, the experiment was performed at different slice locations across the superior sagittal sinus; three slice locations were applied for four volunteers, and six locations were applied for one volunteer.

### Evaluation of linearity between MR signal and slice thickness

**Fig 4.**
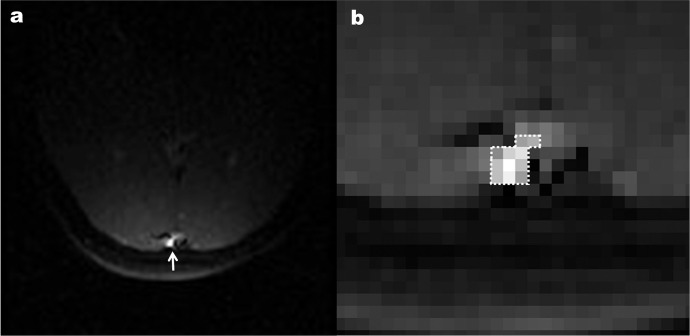
(**a**) SE–EPI image of a slice perpendicular to the superior sagittal sinus (arrow). (**b**) Enlarged view (32 × 32 pixels) of the superior sagittal sinus. The ROI (11 pixels) in the superior sagittal sinus is surrounded by a dotted line.

Figure 4a shows the superior sagittal sinus in an EPI image. Because the receiver gain was automatically adjusted for the slice thickness, we normalized the difference in the receiver gain: the signal intensity of each pixel was divided by the average noise intensity from four regions of interest (ROIs) (100 pixels for each ROI) at the marginal corners of all temporal images of each successive 45 s imaging. Hereafter, the time series data were considered to be normalized. An ROI of 11 pixels (19.4 mm^2^) with the highest signal intensities of the superior sagittal sinus was set as shown in Fig. 4b and applied to all time series images. The signal intensities of the ROI during a 43 s imaging were averaged, excluding the initial eight images (2 s imaging), which were not in the equilibrium state. The linearity between the average signal intensity of the superior sagittal sinus and the slice thickness was evaluated by the determination coefficient (*R*^2^) of the regression line. Whenever the size of the blood vessel remains constant—signifying constant blood flow—for the length of the maximum slice thickness, the average signal intensity increases linearly with an increase in slice thickness. This evaluation was performed for each slice location. The *R*^2^ values were then plotted against the spatial derivative of the blood flow velocity along the blood vessel at each slice location. Data with highly significant *R*^2^ values (>0.77) were used for further analyses, as the data obtained from locations where the blood flow does not change within the maximum slice thickness (15 mm).

### Analysis of the respiratory fluctuation in the MR signal

The time series of the MR signal in the superior sagittal sinus were Fourier-transformed, and the SFI value (Eq. [4]) at the respiratory frequency (0.2–0.5 Hz) was obtained. This analysis was performed for the data from all slice thicknesses. The correlation between SFI and the average signal intensity of the superior sagittal sinus was plotted for all volunteers. Following the aforementioned theory, the gradient of the regression line in Fig. 2, which shows Δ*S/S* in Eq. [7], was obtained from the correlation plot. The blood oxygenation fluctuation (Δ*Y*) was then calculated. The correlation between SFI and the average signal intensity at low (<0.1 Hz) and cardiac pulsation frequencies was also plotted, and Δ*Y* was calculated in each frequency range. The center of the cardiac pulsation range (±0.2 Hz) was set to the individual cardiac pulsation peak around 1 Hz.

### Analysis of the influence of head motion

To assess the influence of head motion, an ROI (16 pixels) was set at the skull near the sagittal sinus on the temporal MR images from one volunteer as a typical dataset. The in-plane head motion appears as a shift in pixel locations in the ROIs. When the head moves by ∆*r* in the imaged slice, the signal in an ROI changes by $\frac{1}{k}\sum_{i=1}^{k}\nabla S_{\text{i}}.\Delta r$ , where *S*_i_ is the signal intensity at each pixel and *k* is the number of pixels in the ROI. Considering the worst case influence of head motion, we assumed that all signal intensity fluctuations at the skull were induced by head motion. Thus, the signal fluctuation due to head motion (*SD*_skull_) can be written as

[8] $$SD_{\text{skull}}=\sqrt{\frac{\sum_{n=1}^{N}{\left(S(n)-S_{\text{average}}\right)}^{2}}{N}}=\left|\frac{1}{k}\sum_{i=1}^{k}\nabla S_{\text{i}}\right|\cdot \left|\Delta r\right|$$

where ∇*S*_i_ is approximated as the vector gradient averaged over the adjacent four pixels. Using *SD*_skull_ obtained from the time series images and the average values of $\left|\frac{1}{k}\sum_{i=1}^{k}\nabla S_{\text{i}}\right|$ of the skull ROI, |Δ*r*| (the standard deviation of the pixel shift due to head motion) was estimated. Following the spectral consideration of Eqs. [3] and [4], the respiratory *SD*_skull_ can be represented by the respiratory *SFI*_skull_, which is calculated from the power spectrum. Inserting the average value of $\left|\frac{1}{k}\sum_{i=1}^{k}\nabla S_{\text{i}}\right|$ in the ROI from the temporal MR images and the respiratory *SFI*_skull_ into Eq. [8] gives |Δ*r*|_r_, which represents the respiratory pixel shift. Provided that Δ*r* denotes in-plane motion, Eq. [8] is also applicable for the ROI at the superior sagittal sinus with the same |Δ*r*|_r_. Thus, the respiratory *SD* at the superior sagittal sinus induced by head motion (*SD*_motion_) is calculated as

[9] $$SD_{\text{motion}}=\left|\frac{1}{k}\sum_{i=1}^{k}\nabla S_{\text{i}}\right|\cdot {\left|\Delta r\right|}_{\text{r}}$$

Taking account into the worst-case scenario of the influence of head motion, we employed the maximum value of $\left|\frac{1}{k}\sum_{i=1}^{k}\nabla S_{\text{i}}\right|$ in each of the time series images to estimate *SD*_motion_ using Eq. [9]. This estimated value of in-plane head motion was compared with the value of the respiratory SFI obtained from the spectrum of the signal fluctuation at the sagittal sinus.

## Results

**Fig 5.**
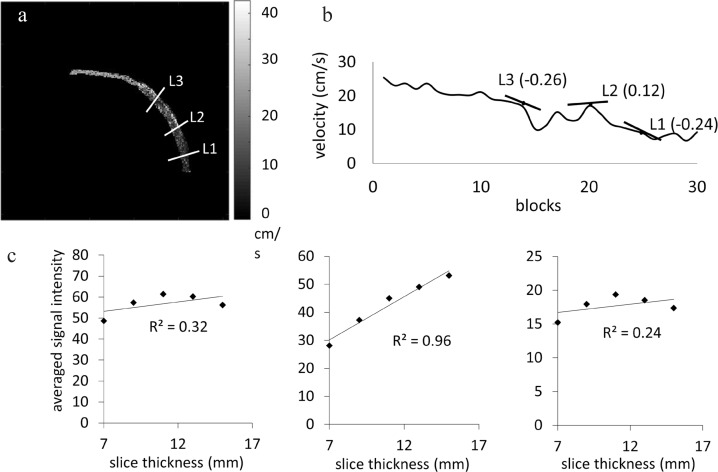
(**a**) Velocity mapping of the superior sagittal sinus of a volunteer. The white lines represent different slice locations. (**b**) Plot of the mean velocity versus block (Fig. 3) along the sagittal sinus of a volunteer. The solid straight lines represent the tangent lines at three-slice locations (L1, L2, and L3 in a). The numbers in parentheses give the derivative of the blood flow velocity along the block. (**c**) Plot of the average signal intensity versus slice thickness at three locations (L1, L2, and L3 from left panel). The solid lines represent the regression lines.

**Fig 6.**
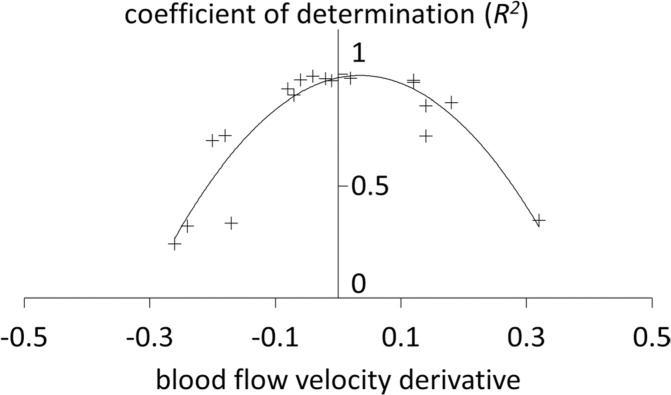
Relationship between the blood flow velocity derivative and the coefficient of determination of the correlation between the average signal intensity and slice thickness. The solid line represents the quadratic regression curve.

Figure 5a shows the velocity mapping of the superior sagittal sinus of a volunteer. The velocity tended to decrease from upstream to downstream, as shown in Fig. 5b. However, the derivative of the blood flow velocity along the block at each slice location in the SE–EPI varied from −0.24 to 0.12 and back to −0.26 at locations L1, L2, and L3, respectively (Fig. 5b). The correlation between average signal intensity and slice thickness increased as the absolute value of the derivative of the blood flow velocity decreased (Fig. 5c). The graph of all volunteers in 18 locations (Fig. 6) shows that the *R*^2^ value of the regression of the average signal intensity and the slice thickness became larger as the absolute value of the velocity derivative decreased. In particular, in the velocity derivative range from −0.1 to +0.1, the average signal intensity and the slice thickness exhibited a significant correlation (*P* < 0.05); in this range, the *R*^2^ values were above the significant criterion (0.77) for the five-slice thickness data.

**Fig 7.**
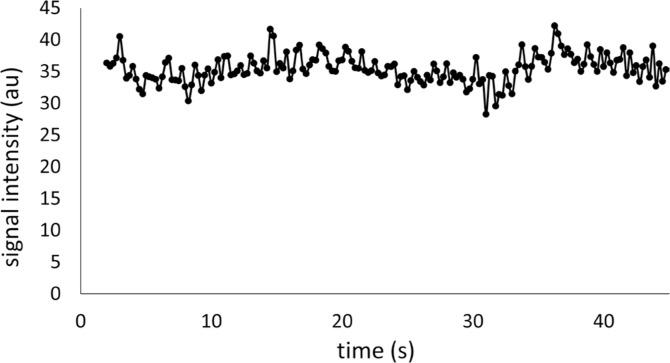
Time series of MRI signal. The signal intensity was divided by the average noise intensity from four ROIs placed at the marginal corners of all images. The transient data of the first eight points, which were excluded from the analysis, are omitted.

**Fig 8.**
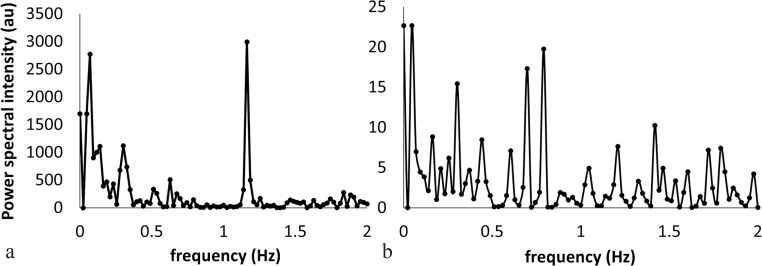
Power spectra of superior sagittal sinus (**a**) and skull (**b**).

After Fourier-transforming the time series of MR signal intensity at the superior sagittal sinus (Fig. 7), the power spectral intensity was obtained (Fig. 8a). This spectrum contained three major peaks, at the low (0.06 Hz), respiratory (0.30 Hz), and cardiac pulsation (1.16 Hz) frequencies. Although the power spectral intensity of the skull also exhibited a number of peaks (Fig. 8b), the intensity was ∼1% of that of the superior sagittal sinus (Fig. 8a).

**Fig 9.**
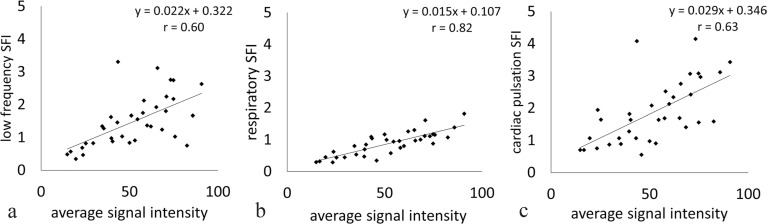
Plots of the (**a**) low frequency, (**b**) respiratory frequency, and (**c**) cardiac pulsation SFI versus the average signal intensity. The solid line in each plot represents the regression line. The data points in each plot were from seven-slice locations of three volunteers: three locations for two volunteers and one location for the other volunteer.

Figure 9 shows the low frequency (a), respiratory (b), and cardiac (c) SFI versus the average signal intensity at slice locations where the derivative of the blood flow velocity was within the range [−0.1, 0.1]. The slopes of the regression lines in these plots are 0.022, 0.015, and 0.029, respectively. All correlations were significant (*P* < 0.01); the correlation coefficients were 0.60, 0.82, and 0.63 for the low, respiratory, and cardiac frequency ranges, respectively. Using Eq. [7], Δ*Y* was calculated to be 1.8 ± 1.0% [mean ± 95% confidence interval (CI)], 1.2 ± 0.3%, and 2.4 ± 1.1% for the low, respiratory, and cardiac frequency ranges, respectively. The y-intercept values, which reflect the fluctuation induced by changes in blood flow velocity, were small: 0.32 ± 0.58 (mean ± 95% CI), 0.11 ± 0.20, and 0.35 ± 0.71 for the low, respiratory, and cardiac frequency ranges, respectively.

As the average value of $\left|\frac{1}{k}\sum_{i=1}^{k}\nabla S_{\text{i}}\right|$ at the skull was 0.64 [1/pixel] and the respiratory *SFI*_skull_ was calculated as 0.062, the value of |Δ*r*|_r_ derived using Eq. [8] was 0.10 [pixel]. At the sagittal sinus, ${\left|\frac{1}{k}\sum_{i=1}^{k}\nabla S_{\text{i}}\right|}_{\text{max}}$ was 0.29 [1/pixel] and *SD*_motion_ was estimated to be 0.053 using Eq. [9]. The respiratory SFI (0.53) calculated from the power spectrum of the signal fluctuation at the sagittal sinus was ten times larger than the estimated *SD*_motion_. As the non-spectral *SD*_skull_ (Eq. [8]) was 0.23, the standard deviation of the non-spectral pixel shift |Δ*r*| was calculated to be 0.36 [pixel].

## Discussion

The evidence that the velocity tended to decrease from upstream to downstream (Fig. 5a, b) indicated a gradual increase in the diametric size of the superior sagittal sinus along with the stream. Because the lowest velocity shown in Fig. 5b is around 8 cm/s, the blood flows at least 20 mm during the TR (250 ms) of SE–EPI used in our experiment. This flow displacement was larger than the maximum slice thickness (15 mm). Moreover, the ROI composed of the maximal 11 pixels in the sagittal sinus was located almost in the center of the cross-sectional sagittal sinus, where the blood flow was faster than near the blood wall. Therefore, the excited blood in the ROI was refreshed every TR for all slice thicknesses used in our experiment, and this inflow effect does not jeopardize our results. Although the average signal intensity of the superior sagittal sinus should increase proportionally with an increase in the slice thickness, this theoretical property was observed where the velocity derivative was small (Fig. 6); the correlation between the average signal intensity and the slice thickness is represented by the value of *R*^2^. At locations, where the derivative of blood flow velocity was small, the diametric size of the superior sagittal sinus was understood to remain almost constant. In the velocity derivative range from –0.1 to +0.1, the average signal intensity showed a significant correlation with the slice thickness. For precise observations of the fluctuation in blood oxygenation in the superior sagittal sinus, the threshold of the absolute value of the velocity derivative may be set to 0.1.

Fluctuations in the signals from the superior sagittal sinus can be observed in the time series of the SE–EPI MR signals (Fig. 7). Its power spectral intensity exhibited three major peaks in the low, respiratory, and cardiac pulsation frequency ranges (Fig. 8a). However, the power spectral intensity of the skull ROI, the intrinsic signal intensity of which was thought to be stable, showed a different type of spectrum (Fig. 8b), indicating the fluctuation induced by head motion. The influence of head motion on the respiratory SFI was evaluated by considering both in-plane and through-plane motion. To evaluate the in-plane displacement during successive SE–EPI (45 s), we focused on the ROI at the skull where the pixel shift due to the displacement of the head caused large signal changes, because the edge of the skull was in the ROI. The respiratory component of the spectra at the skull ROI (Fig. 8b) contains information about the average displacement of the respiratory in-plane head motion (Eq. [8]). Using this information, the spectral intensity of the respiratory in-plane head motion at the sagittal sinus was estimated as 0.053, some 10% of the respiratory SFI (0.53). This evaluation represented the worst-case scenario, because the largest |Δ*r*|_r_ was calculated on the basis that the fluctuation of signal intensity at the skull is all induced by head motion (Eq. [8]), and the maximum spatial signal change in sagittal sinus ${\left|\frac{1}{k}\sum_{i=1}^{k}\nabla S_{\text{i}}\right|}_{\text{max}}$ was used to estimate *SD*_motion_ (Eq. [9]). Therefore, the influence of in-plane head motion, which was <10% of the respiratory SFI at the sagittal sinus, can be neglected. The non-spectral distance of pixel shift due to head motion was estimated to be 0.36 pixels. This standard deviation value of the displacement is equivalent to a peak-to-peak pixel shift of 1.34 (0.36 × $2\sqrt{2}$ × 170/128) (mm). Although this was not especially large, it would be reasonable for a 45 s scan; the posterior side of the head was used as the pivotal point of the head motion, and the head motion around the sagittal sinus and the skull ROI was thought to be small. As for the through-plane head motion, this comes from the shift in the imaged blood vessel of the sagittal sinus. As long as the diameter of the blood vessel is constant, this head motion does not influence the respiratory SFI. As the constancy of the blood vessel was confirmed by the correlation of slice thickness and averaged signal intensity (*see “Evaluation of linearity between MR signal and slice thickness”*), the influence of the respiratory through-plane motion is also neglected in the respiratory SFI at the sagittal sinus. Although the influence of the through-plane motion between the 90° and 180° pulses overlapped on the blood velocity fluctuation, which appears as the y-intersect in Fig. 9, this value of the respiratory SFI was small and does not jeopardize the fluctuation measurement of blood oxygenation (*see “Evaluation of the respiratory fluctuation in blood oxygenation”* in Theory).

The respiratory SFI showed the most significant correlation with the average signal intensity among the major SFIs of low, respiratory, and cardiac frequencies (Fig. 9), indicating that the respiratory fluctuation in venous oxygenation was somewhat stable during our experiments. The venous oxygenation at low frequencies reflected autoregulation^18^ and non-task-related neuronal activities, known as the default mode or functional connectivity.^13^ The metabolic activity of autoregulation^18^ and non-task-related neuronal activity in the resting state modulated the blood flow and oxygen consumption spatiotemporally at low frequencies. Therefore, the amplitude and frequency of venous blood oxygenation at these low frequencies were not as coherent as those at the respiratory frequency, resulting in the worst SFI correlation (Fig. 9a). Pulsatile blood pressure alters the blood flow and venous oxygenation,^19^ according to evidence that venous oxygenation increases with an increase in blood flow under a controlled, stable oxygen consumption in tissue.^20^ Variation in individual blood pressure may have led to the rather low correlation coefficient (0.63) as shown in Fig. 9c. The average signal intensity differed by location, mainly due to the inhomogeneous coil sensitivity of the surface coil used in the experiments.

The fluctuation in venous blood oxygenation at the respiratory frequency in healthy volunteers was calculated to be 1.2 ± 0.3%, whereas those at the low and cardiac pulsation frequencies were 1.8 ± 1.0% and 2.4 ± 1.1%, respectively. Changes in venous blood oxygenation have been reported in the study of functional MRI using phase information of the MR signal^21^ (Fig. 3 indicates that the fluctuation in venous blood oxygenation in a non-active area, which can be considered as a resting state, is <10% of the peak-to-peak value. This value can be converted into the SD value 3.5% (10%/( $2\sqrt{2}$ )) using signal theory^22^ and contains all spectral fluctuation in the venous blood oxygenation). Together with all three major components of our results, the fluctuation in venous blood oxygenation in our experiment was calculated to be 3.3% $\sqrt{{\left(1.8\right)}^{2}+{\left(1.2\right)}^{2}+{\left(2.4\right)}^{2}}$ , which agreed well with the value of <3.5% found in a previous study.^21^

The signal fluctuation due to changes in blood flow velocity should appear as the y-intercept in Fig. 9; y-intercepts of 0.32, 0.11, and 0.35 were found for the low, respiratory, and cardiac frequency ranges, respectively. The validity of the maximum value at the cardiac frequency can be explained as follows. The blood flow velocity in the superior sagittal sinus varies by ±10% over a cardiac cycle.^23^ The average velocity in the superior sagittal sinus at the slice location with a constant blood vessel diameter was calculated to be 97.2 mm/s from the velocity maps of all volunteers, and its peak-to-peak variation of 20% (±10%) led to a 0.29 mm (97.2 mm/s × TE/2 × 20%) variation in refocused signal thickness (gray blocks in Fig. 1). This 0.29 mm cardiac fluctuation can be converted to an MR signal fluctuation (FF) according to the following equation:

[10] $$FF=0.29/(ST-\frac{TE}{2}\times v)\times \frac{SI}{2\sqrt{2}},$$

where *ST* is the slice thickness, *v* is the average blood flow velocity (97.2 mm/s), and *SI* is the average signal intensity in the respective *ST*. The term $ST-\frac{TE}{2}\times v$ represents the thickness of the refocused blood (black blocks in Fig. 1), which produces the MR signal. Because, the calculated cardiac fluctuation (0.29 mm) is a peak-to-peak fluctuation value, $2\sqrt{2}$ was used to convert the peak-to-peak value to an SD value (Eq. [4]) under the assumption of sinusoidal pulsatile variation in the blood flow. The average FF (Eq. [10]) was estimated to be 0.60 from the SI data shown in Fig. 9c, which is within the CI (0.35 ± 0.71) of the y-intercept of the cardiac frequencies. This result indicates the validity of our method. The blood flow velocity fluctuation was due to neurogenic blood volume changes in the arterial side at low frequencies^21,24^ and the central venous pressure at respiratory frequencies.^14,25^ The FFs at low and respiratory frequencies may be understood as equal to or smaller than the cardiac FF from the small y-intercepts found at these frequency ranges.

The fluctuation in venous blood oxygenation at respiratory frequencies (1.2%) was caused by respiratory arteriolar vasomotion driven by respiratory CO_2_ variation in the arterial blood,^10^ and was a marker of cerebral arteriolar vasomotor function. The respiratory center in the brainstem^26^ functions and consumes oxygen at the respiratory frequency, resulting in the respiratory fluctuation of venous blood oxygenation. However, the draining vein from the respiratory center flows into the superior petrosal sinus,^27^ rather than the superior sagittal sinus where we monitored the MR signal. Therefore, the influence of respiratory oxygenation consumption can be disregarded in our experiment. Our method used the physiological fluctuation in PaCO_2_ and is non-invasive, as CO_2_ inhalation, as employed in CVR mapping^8^ to determine arteriolar vasomotor function, was not used. In particular, our newly determined value of 1.2% of the respiratory fluctuation in venous blood oxygenation in healthy volunteers can be considered as a normal index of arteriolar vasomotor function.

## Conclusion

We have successfully demonstrated cerebral venous oxygenation fluctuation at respiratory frequencies in the resting state. This fluctuation was non-invasively evaluated as 1.2% that can be considered as a control value of arteriolar vasomotor function for a healthy human.
